# Immunogenicity of Innovative and Biosimilar Monoclonal Antibodies

**DOI:** 10.3390/antib8010021

**Published:** 2019-03-05

**Authors:** Erik Doevendans, Huub Schellekens

**Affiliations:** Department of Pharmaceutical Sciences, Utrecht University, 3512 JE Utrecht, The Netherlands; h.schellekens@uu.nl

**Keywords:** biopharmaceuticals, monoclonal antibodies, biosimilars, immunogencitity, B-cell tolerance, aggregates, anti-idiotypic

## Abstract

The development of hybridoma technology for producing monoclonal antibodies (mAbs) by Kohler and Milstein (1975) counts as one of the major medical breakthroughs, opening up endless possibilities for research, diagnosis and for treatment of a whole variety of diseases. Therapeutic mAbs were introduced three decades ago. The first generation of therapeutic mAbs of murine origin showed high immunogenicity, which limited efficacy and was associated with severe infusion reactions. Subsequently chimeric, humanized, and fully human antibodies were introduced as therapeutics, these mAbs were considerably less immunogenic. Unexpectedly humanized mAbs generally show similar immunogenicity as chimeric antibodies; based on sequence homology chimeric mAbs are sometimes more “human” than humanized mAbs. With the introduction of the regulatory concept of similar biological medicines (biosimilars) a key concern is the similarity in terms of immunogenicity of these biosimilars with their originators. This review focuses briefly on the mechanisms of induction of immunogenicity by biopharmaceuticals, mAbs in particular, in relation to the target of the immune system.

## 1. General Introduction

The development of the hybridoma technology to produce monoclonal antibodies (mAbs) by Kohler and Milstein counts as one of the major medical breakthroughs of the 20th century. It opened endless possibilities, not only for research, but also to diagnose, prevent, and treat a whole variety of diseases [1].

Initially this discovery led to the introduction of many mAbs in biomedical research and as diagnostic tools relatively fast, but their development as therapeutics was relatively slow. It took 11 years before the murine mAb OKT-3 was officially approved for the prevention of allograft rejection after transplantation [2] and another seven years for the marketing authorization of Reopro to assist percutaneous coronary surgery [3].

There were many reasons why only two mAbs were introduced into the clinic in the 17 years after the development of the technology by Kohler and Milstein. The main problem was that initially only murine derived mAbs were available for clinical use which lack of Fc-functions in humans that are important attributes for, for instance, anticancer activity [4]. However, more importantly, the murine origin was the cause of the high immunogenicity of the first generation of mAbs, which limited the efficacy and was associated with severe infusion reactions [5]. The exact mechanism responsible for infusion reactions caused by any of the mAbs (murine, chimeric, and human) is unclear. Most reactions appear to be the result of antibody antigen interactions resulting in cytokine release.

Several innovations have been introduced in the original hybridoma-based technology by genetic engineering [6]. It enabled the exchange of murine constant parts of the immune globulin chains with the human counterparts resulting in chimeric (murine/human) mAbs. The next step was the introduction of humanized mAbs based on grafting the murine complementary regions (CDR’s) into a human immune globulin backbone.

Transgenic animals expressing the human Ig locus, phage display technologies and different methods to immortalize human B cells allow mAbs based on completely human derived DNA sequences [7]. The expectation was that these human mAbs would be devoid of immunogenicity. However, the claim that “Fully human mAbs are anticipated to be non-immunogenic and thus to allow repeated administration without human anti-human antibody response.” has proven to be false [8].

Humanization has reduced the sometimes extreme immunogenicity associated with murine mAbs, but also the so-called human mAbs have shown to induce antibodies that sometimes have clinical implications [9]. In this chapter we will discuss the possible causes of immunogenicity, the clinical consequences and the assays used to monitor immunogenicity. We will also discuss the issue of immunogenicity of biosimilar mAbs in comparison with the originator medicinal product.

## 2. Immunogenicity of Biopharmaceuticals

The persistence of immunogenicity of human mAbs is no surprise and reflects the experience of over 150 years with biologics as medicines [10]. The first generation of medically used biologics were of animal origin like the antisera produced in farm animals for the treatment of infectious diseases, and like diphtheria and tetanus toxoids that were introduced by the end of the 19th century. In 1921, bovine and porcine insulins became available for the treatment of diabetes and became the most widely used animal proteins in medicine. These products proved to be immunogenic and treatment was sometimes associated with serious immune reactions, like fatal anaphylactic shock and, for example, immune-mediated insulin resistance. Their non-human origin was considered the explanation of their high immunogenicity.

However, the second generation of medically-used biologics which were natural products of human origin introduced in the 50-ties of the last century like growth hormone extracted from human pituitary glands and the plasma derived clotting factors, also proved to be immunogenic in the majority of patients. Their immunogenicity was explained by the lack of immune tolerance for these biologics in the children who needed growth hormone or a clotting factor as substitution because of an inborn deficiency for these proteins.

The introduction of third generation of biologics during the seventies and eighties of the last century, produced by genetic engineering technologies allowing the production of human proteins, like the human insulins, epoetins, interferons, and others intended for use in patients with a normal immune tolerance to these products.

Surprisingly, the great majority of these products appeared to be immunogenic in some patients, with an incidence varying between <1% up to the majority of patients depending on the product. It then became clear that there are two different mechanisms by which these anti-drug antibodies (ADA) are induced by biopharmaceuticals [11]. These two mechanisms also differ in their clinical manifestations.

If the biopharmaceutical is of foreign origin, as is the case with animal derived antisera, the antibody response is comparable to a vaccination reaction. Often a single injection with a “non-human” product is sufficient to induce high levels of neutralizing antibodies. Like the antibodies induced by a vaccine, these antibodies may persist for a considerable length of time. Another hallmark of this type of immunogenicity is the induction of memory cells leading to a booster reaction seen when a patient is re-challenged with the product asparaginase and streptokinase, both of microbial origin, are examples of biopharmaceuticals which are in clinical use today which show this “vaccine” type of immunogenicity.

However, the great majority of biopharmaceuticals are homologues of human proteins of which there is, in general, a high level of immune tolerance in patients. To break B cell tolerance and induce antibodies, prolonged exposure to proteins is necessary. It may take months of chronic treatment before patients start producing antibodies directed against the homologues protein. This type of immune reaction is also milder compared with the immune reaction to non-human proteins. The antibodies are mainly only binding and their clinical effect in most cases is minimal. They disappear relatively quickly when treatment is stopped and there is no memory reaction after re-challenge.

To induce a classical “vaccine-like” immune reaction a degree of non-self is necessary, which is mainly determined by the amino-acid sequence and secondary and tertiary structure of the protein. It is based on the classical activation of the immune system by immune competent cells presenting epitopes of the non-human proteins by their MHC molecules. This activates T cells, which help to activate B cells to produce antibodies. Initially, IgM antibodies of broad specificity and relatively low affinity are formed. By isotype switching and affinity maturation, B cells’ clones will be induced, capable of producing IgG molecules with high affinity as well as memory B cells. As the trigger for this type of immune response is within the structure of the molecule, this immunogenicity can be considered an intrinsic property of the biopharmaceutical.

Basic research, mainly in immune tolerant transgenic mice and in studies with biopharmaceuticals in clinical use showing immunogenicity, indicated that process and product related impurities are triggers for breaking B-cell tolerance [12]. As these triggers are purification and formulation dependent, they are considered as extrinsic immunogenicity. The factors hypothesized to be causing extrinsic immunogenicity include bacterial endotoxins, microbial DNA rich in GC motifs or denatured proteins which all may act as danger signals for the immune system [13].

However, the most convincing extrinsic immunogenic determinant identified is protein aggregation. Apparently aggregates may present as the multimeric array form structures capable of directly interacting with, and activating B cells [14]. This mechanism does not discriminate between self or non-self. It has been shown that also self-antigens are presented in a regular array form with a spacing of 50 to 100 Ångstrom, the B-cell may be activated by dimerization of the B-cell receptor and to start to produce antibodies. Naturally-repeating protein structures are only found in viruses and other microbial agents, suggesting that this type of immune cell activation is old evolutionary mechanisms protecting against infection, preceding the development of the adaptive immune system [15]. Hence, it can be considered as being part of the innate immune system. Box 1 provides an overview of factors potentially contributing to the risk of immunogenicity of biopharmaceuticals.

When tolerance is broken by a biopharmaceutical, the antibody response is often weak with low levels antibodies with low affinity. As it does not need the activity of T-helper cells, isotype switching and affinity maturation is limited and, also, no memory cells are induced. In most cases prolonged treatment is necessary for the antibodies to appear and often the antibody response declines upon further treatment.

This distinction between the vaccine type fulminant immunogenicity reaction and the more restricted and weak antibody response based on breaking tolerance is not absolute: the level of tolerance to proteins, as well as the ability to respond to an immunogen, differ between individual patients. As with many biopharmaceuticals both types of reactions can be seen in the patient population. In hemophilia A the immunogenic response is dependent on the genetic defect in the factor VIII gene [16]. If the defect leads to the complete inhibition of factor VIII expression, the patient will have no immune tolerance resulting in a vaccine like antibody response when treated with factor VIII. However, if the gene defect allows for the expression of sufficient factor VIII with the correct immunogenic make-up to induce tolerance, the antibody response to factor VIII treatment will be predominantly based on breaking tolerance and, in comparison with non-sense mutants, be slow and limited.

Box 1 **Factors contributing to the risk of immunogenicity of biopharmaceuticals.**

**Nature of the Biopharmaceutical**

Size and structural complexity

Sequence variation from endogenous protein

Aggregates

Post-translational & chemical modification (e.g., glycosylation, pegylation).

Neoepitopes due to denaturation or fragmentation

Adjuvant potential of inactive ingredients

Other impurities

 

**Target Disease and Population**

Patient characteristics such as genetic background

Comorbidity

Natural tolerance to protein

Pre-existing immunodeficiency

Use of immunosuppressive drugs or chemotherapy

 

**Treatment Regimen**

Route of administration

Dose

Frequency of treatment

Duration of treatment

## 3. Immunogenicity of Monoclonal Antibodies

The changing pattern of immunogenicity seen during the different steps of humanization of mAbs used resembles the differences seen in immunogenic response of the different generations of biopharmaceuticals [17]. The strong antibody response to the first therapeutic mAbs of murine origin was caused by the intrinsic immunogenicity, the presence of murine “non-self” epitopes in the amino acid sequence. In the following generation of mAbs, the chimeric antibodies, the exchange of the murine constant regions with their human counterparts creating chimeric mAbs resulted in a substantial reduction in immunogenicity. The next generation of therapeutic antibodies is humanized antibodies in which the variable antigen binding regions of the murine mAbs were grafted onto a human monoclonal backbone. However, the reduction in immunogenicity achieved by this additional step in humanization is a matter of debate [18]. The homology between the amino acid sequences of the human and murine variable regions is higher than between their constant regions making a further increase in homology—by humanization of the variable regions—with human antibodies minimal. A comparison of DNA sequence homology of the variable regions of humanized mAbs with human diversity in variable regions sometimes shows more differences than with the murine variable regions [5], or, in other words, based on sequence homology chimeric mAbs are sometimes more “human” than humanized mAbs.

In contrast with the expectations, mAbs completely derived from human sequences (fully human antibodies) proved to be still immunogenic. Thus, other factors than the presence of murine sequences determine the immunogenicity of mAbs. Unlike most other biopharmaceuticals, most therapeutic mAbs (depending on the IgG subclass) have immune modulating activity residing in their Fc parts. Fc functions include macrophage and complement activation, which may boost an antibody response. Removal of N-linked glycosylation at the Fc part of the immunoglobulin reduces these functions and was shown to lead to a diminished immunogenicity [19]. However, the presence of these Fc functions does not completely explain the immunogenicity of human mAbs as antibodies lacking these Fc functions also can be immunogenic. Furthermore, non-human glycosylation, such as galactose-*α*-1,3-galactose, of mAbs produced in mammalian cells, like CHO cells, has been implicated in hypersensitivity reactions. However, these antibodies were not induced by the mAb but were pre-existing “natural”’ IgE antibodies, induced by an endemic tick infection or other pre-exposure galactose-*α*-1,3-galactose, explaining the regional distribution of the hypersensitivity reaction [20].

Antibodies induced by humanized mAbs are predominantly directed to the CDR-regions, which determine their specificity. These anti-idiotypic antibodies may represent the natural antibodies, which, according to the network theory of Jerne are formed to regulate the antibody responses [21]. The anti-CDR antibody response may also reflect a lack of tolerance in individual patients to these epitopes. However, the target of an immune response is not necessarily the part of the molecule that is driving the immune reactions [22].

Whether the immunogenicity of mAbs can also be explained by the extrinsic immunogenicity of monoclonal therapeutic products has not been studied in as much detail as with other biopharmaceuticals, like epoetins and interferons.

There is good experimental evidence about the importance of aggregation. Association between aggregates in immunoglobulin products and immunogenicity (and the induction of tolerance by de-aggregated immunoglobulin products) was already described more than 50 years ago [23]. There are also reports about the induction of an immunogenic reaction towards aggregation of modern monoclonal antibody products in immune tolerant animal models, indicating that breaking tolerance is the main immunological mechanism by which anti-drug antibodies are induced [24].

In addition to the intrinsic and extrinsic immunogenicity of monoclonal antibody products, a number of treatment and patient characteristics may modulate this immune response. An increase in the number of injections and higher doses are associated with a higher immune response, but this seems not necessarily true for all monoclonal antibody products. In some cases, chronic treatment and higher doses have been reported to be less immunogenic than episodic treatment and/or lower dose [25]. The induction of tolerance has been used to explain the reduced induction of antibodies by continuous treatment and by higher doses. These data should, however, be interpreted with caution because under these treatment conditions the level of circulating mAbs is higher and more persistent and the presence of circulating monoclonal antibody during the time of blood sampling for immunogenicity testing may mask the detection of induced antibodies [26].

As with other biopharmaceuticals, the subcutaneous route of administration of mAbs is linked with a higher incidence of immunogenicity than and the intravenous route of administration [27]. Additionally, the immune status of the patients influences the antibody response. Cancer patients are less likely to produce antibodies to biopharmaceuticals, including monoclonal antibody products than patients with a normal immune status. Sometimes immune suppressive agents such as methotrexate are co-administered to patients on monoclonal antibody therapy with the purpose of inhibiting an antibody response [28]. The target of the monoclonal antibody also influences the immunogenic response. In general, products with a cell bound target show a higher level of antibody induction than those with a soluble target. Furthermore, mAbs targeted to immune cells suppress an antibody response.

## 4. Clinical Consequences of Antibodies

Establishing the biological and clinical consequences of immunogenicity of biopharmaceuticals is hampered by both the lack of standardization of the assays and a consensus when to consider a patient antibody positive. This makes it difficult to compare results from different studies and also to develop guidelines about the proper follow-up of antibody positive patients. The antibody response varies greatly between individual patients. A low level of binding antibodies during a short period of treatment has no clinical relevance, but a persisting high level of neutralizing antibodies leads inevitably to a complete loss of efficacy. However, the problem lies in the majority of antibody positive patients showing a response between these two extremes. In diseases like multiple sclerosis and cancer, their unpredictable clinical course and the sometimes relatively modest clinical effects of the biopharmaceuticals are additional hurdles for unambiguously showing loss of efficacy by antibodies.

Antibodies directed to biopharmaceuticals have either no clinical effect, modulate efficacy, cross-neutralize endogenous proteins, or have general immune effects [29]. Induced antibodies may interfere with efficacy in two ways: The antibodies may decrease the efficacy by binding with the target of the biopharmaceutical with higher affinity than the biopharmaceutical or by decreasing their half-life. MAbs distribute mainly present to the main circulation and, therefore, their pharmacokinetic behavior is highly sensitive to the presence of anti-mAbs.

There have been reports that patients making antibodies clinically respond better to therapy with biopharmaceuticals than patients without antibodies. This has been explained by the presence of low affinity antibodies during the initial antibody response extending the half-life of and by increasing the exposure to the therapeutic protein. The enhanced efficacy can also be an epiphenomena: An immunogenic response to a biopharmaceutical could also be a sign of an active immune system contributing to a better response to therapy. Also a better response to the therapeutic effects of mAbs in some cancer patients with antibodies has been reported and was explained by an anti-idiotypic response to the therapeutic monoclonal antibody directed to tumor antigens thereby enhancing the antitumor response [30].

The most serious clinical effect of immunogenicity has been observed with biopharmaceuticals which that are homologous to unique endogenous factors. The antibodies can cross neutralize these (endogenous) factors as has happened with epoetins, which induced neutralizing antibodies neutralizedto erythropoietin, essential for red blood cell maturation, leading to Pure Red Cell Aplasia (PRCA) [31]. Although ADAs directed towards therapeutic monoclonal antibodies, may also cross-neutralize endogenous antibodies, the redundancy in the natural immune response will make a clinical effect difficult to imagine.

The most important clinical effects of immunogenicity of mAbs are infusion reactions (anaphylactoid) and serum sickness [32]. There is a strong association with the level of anti-mAbs and those immune system related side effects of mAbs, which are relatively rare with other biopharmaceuticals. Compared with other biopharmaceuticals, mAbs are injected/infused in relatively high amounts in the circulation, which may result in formation of high levels of immune complexes, as a consequence of immunogenicity.

## 5. Assays for Antibodies Induced by Monoclonal Antibodies

The standard approach for detecting antibodies induced by biopharmaceutical in sera of patients is to first screen with a highly sensitive assay for binding antibodies [33]. This assay should have a cut off at the 5% false positivity rate. To discriminate between real and false positives, the specificity of the binding is evaluated by a displacement assay. The biopharmaceutical is added to serum and if this leads to a significant reduction of the signal, the serum is qualified as true positive. The antibody response is then further characterized, for instance, by titrating the antibody level, determining the isotypes of the antibodies involved, and check whether the antibodies are neutralizing.

The preferred format for screening for binding antibodies is the bridging assay. In this type of assay the biopharmaceutical is used to capture the antibodies present in the patient sera and the captured antibodies are detected by adding labeled biopharmaceutical as a probe. Such bridging assays are independent of the type of antibodies to be detected, enabling the use of antisera induced in animals as a positive control. The same assay can be used for the determination of antibodies in treated patients, as well as in animal studies. Since the bridging assay only detects binding by proteins with double binding sites, it is more specific than the standard ELISA type of binding assay. However, the bridging immune assay may miss low affinity IgM type of immune response because of the washing steps involved. Therefore, for detection of early immune responses, biosensors applying surface plasmon resonance technology are advocated instead of ELISA type assays. In addition, it may be important to assay for the presence of neutralizing antibodies [34], which may interfere with the biological and clinical activity of the biopharmaceutical. Assays for neutralizing activity are based on the inhibition of a biological effect of the biopharmaceutical in vitro, Assays for neutralizing activity need to be designed for each biopharmaceutical individually and are inherently difficult to standardize because every biopharmaceutical has its own specific biological effect measured by a specific bioassay. In cases where there is no bioassay available, the possible neutralizing effect of the induced antibody can be assayed by testing whether the anti-drug antibodies inhibit the binding of the biopharmaceutical with its target.

Any assay for antibodies induced by biopharmaceuticals is sensitive for drug circulating at the time of sampling which may interfere with the assay. This is especially the case for therapeutic because of their relative long half-life of, the presence of natural antibodies, receptors, and immune complexes, which all may interfere with assay results and be the cause of false negative results.

Over the years, many drug-tolerant assay formats have been developed to measure induced antibodies in the presence of large amounts of drug [35]. Antibodies forming complexes with the drug are difficult to detect. Most drug-tolerant assays use a form of acid treatment step to dissociate the antibodies from the drug. Subsequently, the excess drug is captured or removed or a substantial amount of labeled drug is added that will compete with drug in the sample. Then the free antibodies can be detected. These protocols can be used both for binding as well as bioassays. Potential drawbacks of acid treatment are a significantly higher background, loss of sensitivity due to damaged antibodies, or release of that may interfere in bridging assays and give rise to false-positive results.

Several new techniques to measure induced in the presence of drugs have been developed in the last few years. An example is the affinity capture elution ELISA (ACE) in which the induced antibodies from acidified serum samples are captured by immobilized drug and the excess of drug is washed away. In a second acidification step, the antibodies are released and absorbed onto a second carrier and detected in an electro-chemo-luminescence (ECL) bridging assay. Other examples of test formats are the biotin-drug extraction with acid dissociation (BEAD) assay the sample pre-treatment bridging ELISA, the acid dissociation radioimmunoassay, the temperature-shift radioimmunoassay, and the homogeneous mobility shift assay (HMSA).

Due to the difficulties in their validation, in medical practise these assays are hardly used for clinical decision-making. As alternative with mAbs, drug trough levels are being measured as a marker for clinical activity of the drug [36].

## 6. Immunogenicity and Biosimilar Monoclonal Antibodies

The potential immunogenicity of biopharmaceuticals was one the main reasons behind the dedicated regulatory biosimilar pathway for copy products after the patents and market exclusivity of the original biopharmaceuticals expire. The main difference with the generic pathway for copies of small molecules is the need for clinical trials. Biopharmaceuticals were considered too complex and heterogeneous to be completely characterized. Additionally, original products and their copies could, therefore, never be shown to be identical. To get a marketing authorization as a biosimilar, the copy needs to be similar in physical-chemical and biological characteristics and this similarity needs to be confirmed by (pre)clinical studies. In these clinical studies the immunogenicity between the original and biosimilar candidate always needs to be studied. The need for clinical to evaluate the immunogenicity is based on the notion that immunogenicity is closely linked to product characteristics as glycosylation and impurities in which the biosimilar and reference product are most likely to differ.

There have been 18 biosimilar mAbs authorized in the EU by September 2018. Biosimilars and original biopharmaceuticals share the same amino acid sequence and their secondary and tertiary structure needs to be similar. So the intrinsic immunogenicity of the biosimilar will be comparable with the original product. If there is a difference between the two, it will most likely caused by differences in the extrinsic immunogenicity, like the level of impurities mainly aggregates.

In Table 1, the relative immunogenicity of biosimilars and their reference products are listed and including the differences in impurities and glycosylation. These data are derived from the European Public Assessment Reports available on the EMA website [37]. The observation with these monoclonal biosimilars confirm that there are always small differences between biosimilars and original products, mainly concerning glycosylation. But these differences have apparently no impact on immunogenicity, which proved to be comparable in all cases. This confirms data from other biopharmaceuticals and it is likely that also with mAbs aggregation is the most important driver of the extrinsic immunogenicity.

## Figures and Tables

**Table 1:** Relative immunogenicity of biosimilar monoclononal antibodies.

Brand Name Original	Manufacturer Brand Name(s) Company Code	Structural Differences	ADA Assays Format Binding Ab Neutralizing Ab	Pivotal Trial Indication Dose Route	Number of Patients Length of Treatment	Immunogenicity Results	Remarks
Adalumimab Humira	**Sandoz** Halimatoz Hefiya GP 2017	> N glycans >galactosylation >non fucosylation < high mannose	ECL bridging assay Ligand binding assay	Psoriasis 40 mg Subcutaneous	231 GP 2017 234 Humira Up to 49 weeks	No difference	Nearly all neutralizing antibodies
	**Amgen** Amgevita Solymbic ABP 501	Minor quantitative differences in glycan structures	ECL Bridging assay Ligand binding assay [38] *	Psoriasis 40 mg biweekly Subcutaneous RA 40 mg biweekly Subcutaneous	175 ABP 501 175 Humira Up to 52 weeks 264 APB 501 262 Humira Up to 26 weeks	No difference	About 1/3 neutralizing
	**Boehringer** Cyltezo BI695501	Differences were observed	ECL bridging assay ADCC inhibition	RA 40 mg biweekly Subcutaneous	324 BI695501 321 Humira 48 weeks	Comparable immunogenicity	About 50% neutralizing
	**Samsung** Imraldi SB5	%G0F, %Afucose, %sialylation Charge variants slightly different	bridging ligand-binding (ECL) inhibition of TNF-α binding to SB5	RA 40 mg Biweekly Subcutaneous	271 Imraldi 273 Humira 52 weeks	Comparable immunogenicity	About 100% neutralizing
Rituximab MabThera Rituxan	**Celltrion Blitzima** Truxima Rituzema Ritemvia CT-P10	Some slight differences	state-of-art and validated immunoassays	RA Up to 6 infusions of 1 g	161 CT-P10 211 MabT/Ritu 72 weeks	Comparable however antibodies to CT-P10 appeared earlier	
	**Sandoz** Rixathon Riximyo GP2013	Slightly higher purity Lower level of acidic variants Minor differences in glycosylation	ELISA and ECL for binding Validated assay for neutralization	RA 2–4 infusions of 1 g Follicular Lymphoma 8–16 cycles of 375 mg/m^2^ infusions	86 GP2013 87 MabThera 52 weeks 268 GP 2013 283 MabThera 3 years	Very low incidence but comparable	
	Pfizer/Celltrion Inflectra Remsima	Slightly higher levels of aggregates (higher levels of G1FNeuGc and G2F1NeuGc difference in the level of afucosylated glycans,	An electrochemiluminescent (ECL) immunoassay competitive ligand binding assay.	RA 3mg/kg iv Every eight weeks	302 CT-P13 304 Remicade 54 weeks	No marked differences	All antibodies neutralizing
	Sandoz Zessly	Differences in charge heterogeneity some minor/ trace glycoforms show differences	ECL bridging assay single cell-based NAb assay strategy	RA 3 mg/kg iv Every eight weeks	280 Zessly 286 Remicade 78 weeks	No differences	About 50% neutralizing

* ADA assay formats reported for ABP 501 and Amjevita only.
